# Clinical phenotypes and outcomes of spinal cord stimulation in painful peripheral polyneuropathy: a cohort including diabetic, chemotherapy-induced, and idiopathic etiologies

**DOI:** 10.3389/fpain.2026.1954493

**Published:** 2026-10-07

**Authors:** Ángeles Canós, Beatriz Castel, Ara Bermejo, Rosa María Izquierdo Aguirre, Ruth Robledo, Elisa Gallach, Teresa Sevilla, Pilar Argente, Ismael Huertas

**Affiliations:** 1 Multidisciplinary Pain Management Unit, La Fe University and Polytechnic Hospital, Valencia, Spain; 2 Department of Anaesthesiology, La Fe University and Polytechnic Hospital, Valencia, Spain; 3 Department of Neurology, Valencia University and La Fe Polytechnic Hospital/IISLAFE/CIBERER, Valencia, Spain; 4 Neuromodulation R&D, Boston Scientific Corporation, Valencia, CA, United States.

**Keywords:** autonomic dysfunction, chronic polyneuropathy, pain, peripheral neuropathy, spinal cord stimulation

## Abstract

**Background and objective:**

Chronic painful peripheral polyneuropathy (PPN) is a leading global cause of chronic pain and may arise from a wide range of etiologies. When pharmacological treatments fail to provide adequate pain relief, spinal cord stimulation (SCS) has demonstrated efficacy in selected subtypes. This study investigated the effectiveness of SCS in alleviating pain, neurological symptoms and signs, and other health-related outcomes in patients with diverse forms of PPN, including diabetic (DPN) and chemotherapy-induced (CIPN) peripheral neuropathies, and chronic idiopathic axonal polyneuropathy (CIAP).

**Methods:**

This prospective, single-center observational study included 30 patients with drug-refractory PPN (11 DPN, 8 CIPN, 9 CIAP, and 2 other etiologies: 1 amyloidosis and 1 Fabry disease). All patients were implanted with two percutaneous leads at T10–T11 targeting lower limbs; a cervical lead (C4–C7) when upper limb pain was present; and SCS system. Low-frequency (<100 Hz) sub-perception and/or supra-perception stimulation modalities were offered. Clinical outcomes were assessed at baseline and at 2, 4, 6, and 12 months post-implantation and included pain (VAS, SF-MPQ-2), neuropathic symptoms (NPSI), autonomic symptoms (SAS), neurological signs, and other health-related factors. Intraepidermal nerve fiber density (IENFD) was assessed at baseline and at 12 months.

**Results:**

Baseline evaluations demonstrated severe disease burden, with CIAP patients exhibiting more pain symptoms and DPN patients more neurologic deficits. SCS produced a significant and sustained reduction in pain intensity and symptoms (VAS from 8.6 to 2.0, SF-MPQ-2 from 115.5 to 23.0; *p* < 0.001) across all PPN subtypes, with greater improvements observed in DPN (*p* < 0.001). Additional benefits included reductions in neuropathic symptoms (NPSI from 61.1 to 11.1; *p* < 0.001), autonomic symptoms (SAS from 16.5 to 9.9; *p* = 0.02) and improvements in sleep, functioning, and quality of life (EQ-VAS from 38.8 to 77.9; *p* < 0.001). Neurological signs remained unchanged. Use of analgesics decreased substantially (59% complete withdrawal; 41% partial), and 96% of patients reported “much” or “very much” improvement. Twelve-month IENFD measurements showed a lower-than-expected mean decline across etiologies, with IENFD increases outnumbering decreases, although the changes were modest.

**Discussion:**

SCS was associated with substantial and durable improvements in pain, neuropathic and autonomic symptoms, and other wellbeing outcomes across multiple painful PPN etiologies. The IENFD results suggest that SCS may have some effects on small-fiber integrity.

## Introduction

Peripheral neuropathy is a common neurological disorder encompassing a heterogeneous group of diseases with over 100 identified causes and risk factors. It can be classified according to the number of nerves affected (mononeuropathy vs. polyneuropathy), temporal course (acute vs. chronic), or underlying pathological process (axonal vs. demyelinating). Among these, chronic peripheral polyneuropathy (PPN) represents one of the most prevalent forms and includes a broad spectrum of etiologies, such as metabolic disorders, immune-mediated diseases, toxic exposures, and hereditary conditions (1–3). In approximately 25%–30% of cases, no underlying cause can be identified, and the condition is classified as idiopathic (4).

The prevalence of PPN is estimated at approximately 7% in the elderly population and 1%–3% in the general population of developed countries (5). Notably, it represents one of the leading causes of chronic pain worldwide. Diabetic peripheral neuropathy (DPN), one of its most common subtypes, affects up to 50% of individuals with diabetes, with approximately one-third developing painful symptoms (6).

Clinically, patients typically present with symmetrical sensory symptoms affecting extremities, including numbness, paresthesia, muscle weakness, and pain. The disease generally follows a gradual onset and slow progression, with a characteristic length-dependent pattern that begins distally and progresses proximally (7). The pain is typically described as burning in nature and may worsen at night, thereby disrupting sleep. Allodynia and muscle cramps may be also present. Neuropathy often disrupts the autonomic nervous system, affecting cardiovascular, gastrointestinal, urogenital, and sudomotor functions and leading to symptoms such as abnormal sweating, orthostatic intolerance, and gastrointestinal and urogenital disturbances (8) (Figure 1).

**Figure 1:**
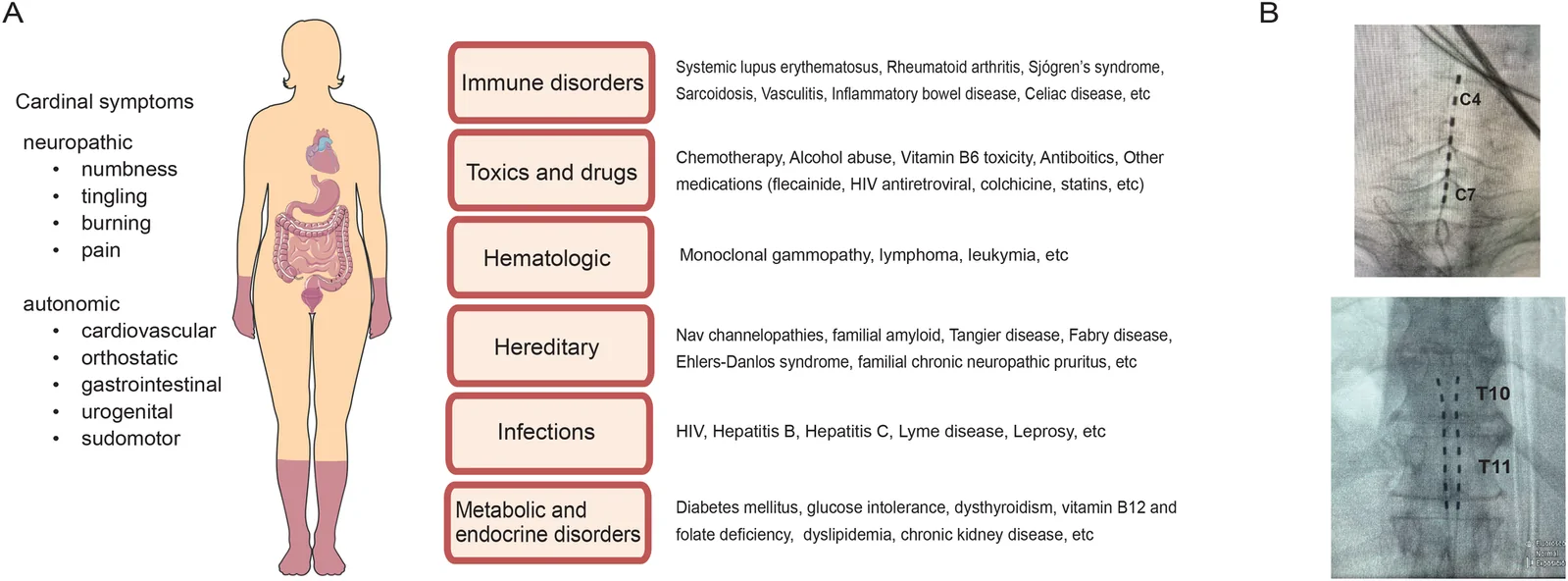
Clinical presentation and etiology classification of polyneuropathy along with spinal cord stimulation leads placement. **(A)** Classical clinical presentation and etiological classification of chronic polyneuropathy, adapted from Nolano et al., 2020 (3), illustrating length-dependent somatic neuropathy and autonomic dysfunction. **(B)** Epidural Spinal Cord Stimulation (SCS) lead placement at thoracic and cervical vertebral levels to treat lower and upper limb pain.

The pathophysiological hallmark of PPN is nerve injury preferentially affecting small nerve fibers (i.e., thinly myelinated A*δ* fibers and unmyelinated C fibers), which are present in both the somatic and autonomic nervous systems (9). Some polyneuropathies may exhibit a mixed phenotype, involving both small and large nerve fibers. The gold standard for diagnosing small fiber involvement is the quantification of intraepidermal nerve fiber density and/or thermal threshold testing, as routine electromyography and nerve conduction studies primarily detect large fiber dysfunction. In practice, diagnosis is often based on the presence of otherwise unexplained neuropathic pain and autonomic symptoms, together with physical examination findings such as reduced pinprick and temperature sensation.

Current medical management is primarily focused on symptom relief, particularly pain control, through pharmacological therapies. Commonly prescribed medications include anticonvulsants (e.g., gabapentin, pregabalin), antidepressants (e.g., duloxetine, amitriptyline), and opioids (10). However, these treatments often provide limited or non-sustained efficacy, resulting in persistent patient burden. It has been estimated that up to 50% of patients with DPN discontinue these medications within three months of initiation (11).

Spinal cord stimulation (SCS) is a well-established and widely used therapy for chronic pain and has demonstrated efficacy in patients with polyneuropathy who are refractory to conventional medical management. Initial evidence dates back to the 1990s, with a case series of nine patients with painful DPN (pDPN) (12). Since then, the evidence has expanded, with randomized controlled trials demonstrating robust efficacy in pDPN (13, 14). However, evidence for other PPN subtypes remains limited. Case reports have suggested potential benefit in chemotherapy-induced peripheral neuropathy (CIPN) (15). CIPN is estimated to account for approximately 15% of PPN cases (1), and its incidence is expected to increase with improved cancer survival rates. In contrast, evidence for chronic idiopathic axonal polyneuropathy (CIAP) (4) remains lacking, despite this subtype potentially representing up to one-third of PPN cases (1).

The aim of this study was to evaluate the effectiveness of SCS across a broader spectrum of chronic painful PPN. We included patients with three of the most common subtypes—DPN, CIPN, and CIAP—as well as cases with less frequent etiologies. Our objectives were to comprehensively characterize these patients, assess potential clinical differences among subgroups, and evaluate the effects of SCS on key clinical outcomes, including pain, neurological signs and symptoms, autonomic dysfunction, and aspects of daily living such as health-related quality of life, sleep, and functioning. Building upon previous preliminary findings, this manuscript presents updated data from an expanded cohort, including longer-term follow-up and more detailed analyses.

## Methods

### Study design and participants

This was a prospective study conducted in La Fe University and Polytechnic Hospital, Valencia, Spain. Candidate patients who provided informed consent were screened for eligibility according to predefined inclusion and exclusion criteria. Key inclusion criteria were: (i) age ≥18 years; (ii) clinical diagnosis of chronic polyneuropathy for more than 1 year; (iii) pain refractory to pharmacological treatment for at least 1 year, including gabapentinoids; (iv) morphine dose or equivalent <120 mg/day; (v) pain score >5; (vi) body mass index (BMI) < 45; (vii) HbA1c < 10% (for patients with DPN); and (viii) no prior spinal cord stimulation for any indication. Key exclusion criteria included: (i) patient immunocompromised or with a history of cancer requiring active treatment within the previous 6 months; (ii) documented history of substance abuse (e.g., narcotics, alcohol) or dependence within the past 6 months; (iii) active systemic infection; (iv) pregnancy; (v) participation in another clinical study involving an active treatment arm; (vi) severe neurological or psychiatric disorders; (vii) ongoing anticoagulant therapy; and (viii) active skin infection at the planned site of device implantation.

Prior to implantation, and in accordance with standard practice at our center, all patients attended an on-site educational session on SCS led by a clinical psychologist (E.G.). Following this session, patients could decide whether or not to proceed with implantation.

### Standard protocol approvals, registrations, and patient consents

The study was conducted in accordance with the Code of Ethics of the World Medical Association (Declaration of Helsinki) for experiments involving humans. All procedures were performed in compliance with relevant laws and institutional guidelines and have been approved by the Research Ethics Committee of La Fe University and Polytechnic Hospital, Valencia, Spain (Registration number: 2022-663-1). All patients agreed to participate in the study and accepted and signed informed consent. This study adhered to European and Spanish laws concerning personal data protection.

### Spinal cord stimulation

Patients who met eligibility criteria underwent SCS trial and implantation procedures in accordance with standard practice at our center. Subjects presenting with lower limb pain only were implanted with two 8-contact percutaneous epidural leads positioned at the T10–T11 vertebral levels. Subjects with both lower and upper limb pain additionally received a third 8-contact lead placed at the C4–C7 levels (Figure 1B). Intraoperative paresthesia testing was performed to ensure adequate coverage of the painful regions, including the most distal areas. A trial phase of 7–14 days using an external stimulator was then conducted. Patients achieving ≥50% pain relief proceeded to permanent implantation with an implantable pulse generator (IPG) (WaveWriter Alpha™, Boston Scientific).

SCS programming was individualized based on patient preference for either the absence (sub-perception) or presence (supra-perception) of stimulation-induced paresthesia. The available stimulation modalities included “fast-acting sub-perception therapy” (FAST^TM^) and “combination therapy” (COMBO^TM^). The FAST modality consists of a paresthesia-guided targeting via a high-resolution electrical field shaped with multiple independent current control, delivered at 90 Hz using symmetric biphasic pulses, pulse-width of 150–300 µs and sub-perception amplitude at ≤50% of perception threshold (PT) (16). Combination therapy integrates two or more stimulation sites and modalities: (i) conventional paresthesia-based stimulation delivered at low frequency (40–80 Hz), with a pulse-width of 200–400 µs and supra-perception amplitude (∼100%–120% of PT); and (ii) an anatomically guided electrical configuration (Contour^TM^) designed to maximize modulation of dorsal horn elements over dorsal columns (17), delivered at 50 Hz with symmetric biphasic pulses, a pulse-width of 300 µs, and sub-perception amplitude at ≤50% of PT.

### Clinical assessments

Demographic information, medical history, and stimulation parameters were collected for all patients as part of their routine follow-up. Clinical assessments were performed at baseline and post-implant (2, 4, 6, and 12 months after implant) by certified physicians: a pain specialist (B.C.) and a neurologist/rehabilitation specialist (A.B.). Pain assessments included evaluation of pain intensity using the Visual Analog Scale (VAS), as well as the following questionnaires: Douleur Neuropathique-4 (DN4); the Neuropathic Pain Symptoms Inventory (NPSI): a 12-item questionnaire (10 severity+2 frequency items) covering five neuropathic symptoms' domains (burning, pressing, paroxysmal and evoked pain, and paresthesia/dysesthesia) (18); and the Short-Form McGill Pain Questionnaire-2 (SF-MPQ-2): a 22-item questionnaire covering four pain domains (continuous, intermittent, neuropathic, and affective pain) (19).

Autonomic symptoms were evaluated using the Small Fiber Neuropathy Symptoms Inventory Questionnaire (SFN-SIQ): a 13-item frequency-based questionnaire (scored 0–3) combining 10 autonomic and 3 sensory items (20), and the Survey of Autonomic Symptoms (SAS): a 12-item questionnaire assessing how bothersome (scored 1–5) common autonomic symptoms are (21).

Neurological signs were assessed using the Utah Early Neuropathy Scale (UENS) (22), which comprises several physical tests evaluating sensory function (pin sensation, large fiber sensation and allodynia), motor function (great toe extension), and the deep tendon reflex. Skin punch biopsies from the distal leg and proximal thigh were collected at baseline and 12 months post-SCS to assess changes in intraepidermal nerve fiber density (IENFD).

Additional health and well-being outcomes included: (a) health-related quality of life (HR-QoL) by the VAS and utility index from 5-level EuroQol 5 Dimensions (EQ-5D-5L); (b) pain-related sleep interference by the 3-item Chronic Pain Sleep Inventory (CPSI-3) (23); and (c) functioning by the Global Assessment of Functioning (GAF) (24). Changes in analgesic medication were recorded, along with the Global Impression of Change following treatment, as reported by both the clinician (C-GIC) and the patient (P-GIC). Complications and adverse events were also recorded.

### Statistical analyses

Descriptive statistics were calculated for all study variables and are presented as frequencies and percentages for categorical variables and as mean ± standard deviation for continuous variables. Distributional assumptions for continuous variables were assessed using the Shapiro–Wilk test together with visual inspection of data distributions.

Baseline comparisons between etiology groups were performed using one-way analysis of variance (ANOVA) for continuous variables and the chi-square test for categorical variables, with appropriate *post-hoc* comparisons performed when applicable.

Longitudinal changes in the main quantitative outcomes following SCS were analyzed using linear mixed-effects models (LMM), including time as a categorical fixed effect and participant as a random effect to account for within-subject correlations across repeated measurements. Age and sex were included as covariates. Additional LMMs incorporated etiology and time-by-etiology interaction as fixed effects to evaluate whether longitudinal changes differed between PPN subtypes.

Item-level symptom analyses were performed using paired t-tests comparison between baseline and the last visit. Changes in paired categorical variables were assessed using the McNemar test. Statistical significance was defined as *p* < 0.05. Given the exploratory nature of the study, no adjustment for multiple comparisons was applied. All analyses were performed using IBM SPSS Statistics.

## Results

### Subjects

Over a three-year period, a total of 44 patients with chronic polyneuropathy (PPN) referred to our unit were deemed eligible candidates. Of these, 8 did not proceed to implantation (6 DPN withdrew consent, and 2 CIPN were excluded due to medical contraindications related to cancer progression), and 1 DPN did not pass pre-surgical screening.

Thus, 35 patients underwent an SCS trial. Of these, 30 had a successful trial and were permanently implanted (11 DPN, 8 CIPN, 9 CIAP, and 2 with other etiologies: 1 amyloidosis and 1 Fabry disease), while 5 (3 CIPN and 2 CIAP) did not surpass trial (trial-to-perm ratio: 86%).

The mean age was 60 years (range 33–80), and 20/30 patients (67%) were male. As commonly observed in SCS studies, the mean pain duration was long: 8.2 years (range 1–22). Among the implanted patients, 18 (60%) reported pain confined to the lower limbs and were therefore implanted with two thoracic leads, whereas 12 (40%) had pain in both lower and upper limbs and were implanted with dual thoracic and a cervical lead.

The complication rate was low. One patient with CIPN was excluded after 3 months due to atypical clinical evolution (erythromelalgia with rapid and severe progression requiring rescue treatment with intravenous immunoglobulin). Another patient required explantation due to infection, and one additional patient underwent lead revision.

### Baseline characteristics

Baseline assessments demonstrated substantial disease burden across sensory, autonomic, and health-related domains (Table 1). Pain severity was high, with mean pain intensity of 8.6/10 (VAS), neuropathic symptom burden of 7.0/10 (DN4) and 61.1/100 (NPSI), and multidimensional pain severity of 115.5/220 (SF-MPQ-2). All pain measures fell within the “severe” range according to their respective severity classifications. Autonomic involvement was also evident, in the moderate range, with mean symptom scores of 13.5/39 on the SFN-SIQ and 16.5/60 on the SAS.

**Table 1:** . Baseline characteristics for all patients and stratified by etiology, along with statistical subtype comparisons.

Outcome	All (30)	Subtype comparisons			
		DPN (11)	CIPN (8)	CIAP (9)	*P*-value
Demographics					
Sex (M/F)	20/10	8/3	4/4	6/3	0.54
Age (SD)	60.5 (12.5)	64.5 (10.0)	56.6 (12.8)	59.4 (16.3)	0.83
Pain duration	8.2 (5.6)	9.3 (5.1)	3.8 (2.0)*,***	10.2 (7.1)	0.04
Pain					
VAS	8.6 (0.9)	8.5 (0.9)	8.6 (1.1)	8.7 (0.9)	0.90
DN4	7.0 (1.3)	6.6 (1.6)	7.0 (1.3)	7.2 (1.0)	0.60
NPSI	61.1 (20.9)	52.2 (20.8)	67.9 (20.4)	64.8 (20.8)	0.21
SF-MPQ-2	115.5 (42.4)	98.2 (38.7)	123.6 (34.1)	129.0 (50.6)	0.25
Autonomic					
SFN-SIQ	13.5 (5.4)	12.4 (5.5)	14.8 (3.7)	12.6 (6.2)	0.59
SAS	16.5 (8.6)	16.3 (8.0)	14.5 (8.8)	17.2 (10.1)	0.82
HR-QoL					
EQ-index	0.70 (0.06)	0.71 (0.06)	0.69 (0.07)	0.69 (0.04)	0.70
EQ-VAS	38.8 (18.0)	34.5 (12.6)	37.1 (14.1)	46.7 (23.0)	0.20
Sleep					
CPSI-3	18.4 (8.3)	18.4 (9.0)	20.7 (6.9)	16.7 (9.8)	0.64
Functioning					
GAF	63.9 (9.4)	63.2 (7.6)	69.5 (5.0)	60.7 (12.6)	0.14
Neurological					
UENS	17.3 (6.5)	20.3 (6.1)	16.4 (4.6)	13.3 (6.0)*	0.04
Motor	2.8 (1.8)	2.8 (1.9)	2.3 (2.1)	2.8 (1.7)	0.94
Pin sensation	6.5 (4.7)	9.1 (4.0)**,***	4.4 (4.0)	4.7 (5.0)	0.05
Allodynia	1.7 (0.7)	1.6 (0.8)	1.7 (0.8)	1.7 (0.7)	0.92
Large fiber sensation	3.3 (1.9)	3.6 (2.1)	3.7 (2.1)	2.2 (0.7)	0.13
Deep tendon reflex	3.0 (1.4)	3.2 (1.4)	3.7 (0.8)	2.0 (1.4)**	0.02
IENFD_proximal_ *(n* *=* *22)*	5.6 (2.4)	4.6 (2.1)	5.9 (3.1)	6.1 (2.2)	0.45
IENFD_distal_ *(n* *=* *22)*	3.0 (2.1)	2.2 (1.2)	3.5 (2.5)	3.1 (2.7)	0.58

DPN, diabetic peripheral neuropathy; CIPN, chemotherapy-induced peripheral neuropathy; CIAP, chronic idiopathic axonal polyneuropathy; VAS, visual analog scale; DN4, douleur neuropathique-4; NPSI, neuropathic pain symptoms inventory; SF-MPQ-2, short-form mcgill pain questionnaire-2; SFN-SIQ, small fiber neuropathy symptoms inventory questionnaire; SAS, survey of autonomic symptoms; HR-QoL, health-related quality of life; EQ, EuroQol; CPSI-3, 3-item chronic pain sleep inventory; GAF, global assessment of functioning; UENS, Utah early neuropathy scale; IENFD, IntraEpidermal nerve fiber density.

*P*-value: statistical significance (*p* < 0.05) in ANOVA

*p < 0.05 in pairwise comparison vs. DPN

**p < 0.05 in pairwise comparison vs. CIPN

***p < 0.05 in pairwise comparison vs. CIAP

HR-QoL was highly impaired, with mean EQ-VAS and EQ-index scores of 38.8/100 and 0.70/1, respectively. Pain-related sleep interference was substantial (CPSI-3: 18.4/30), and functioning was also reduced (GAF: 63.9/100).

Neurological assessments confirmed substantial large- and small-fiber involvement across the cohort. The mean UENS total score was 17.3, consistent with moderate-to-severe neuropathy. Deficits were observed across sensory domains, including pin sensation (6.5/24), large-fiber sensation (3.3/8), and allodynia (1.7/4), as well as motor function (2.8/4) and deep tendon reflexes (3.0/4). Among patients with available IENFD (22/30), small-fiber involvement was confirmed, with reduced IEFND at distal-leg (3.0 fibers/mm) and proximal-thigh (5.6 fibers/mm) compared with normative values (4.5 and 8.5 fibers/mm, respectively). Overall, baseline findings indicated a predominantly mixed neuropathy phenotype involving both small- and large-fiber injury, although more prominent in DPN and CIPN than in CIAP.

There were some baseline characteristics that differed across PPN subgroups (Table 1). Pain duration was shorter in patients with CIPN compared with DPN and CIAP (3.8 vs. 10.2 years). Pain intensity was comparable between groups (VAS), whereas multidimensional pain measures showed higher, yet not significant, symptom burden in CIPN and CIAP compared with DPN (e.g., SF-MPQ-2: 98 vs. 126). Subdomain analyses demonstrated higher evoked pain (hyperalgesia, allodynia) in CIPN and CIAP compared with DPN (6.86 and 5.11 vs. 1.20, respectively), together with greater burning feet and affective pain components (Figure 2A). The patient with Fabry disease had 14 years of pain duration, high pain intensity (VAS = 9) and moderate burden of pain components (SF-MPQ-2: 80, NPSI = 45) and autonomic symptoms (SAS = 16). The patient with amyloidosis had 8 years of pain duration, high pain intensity (VAS 9.5) and severe burden of pain components (SF-MPQ-2: 155, NPSI = 88) and autonomic symptoms (SAS = 28).

**Figure 2:**
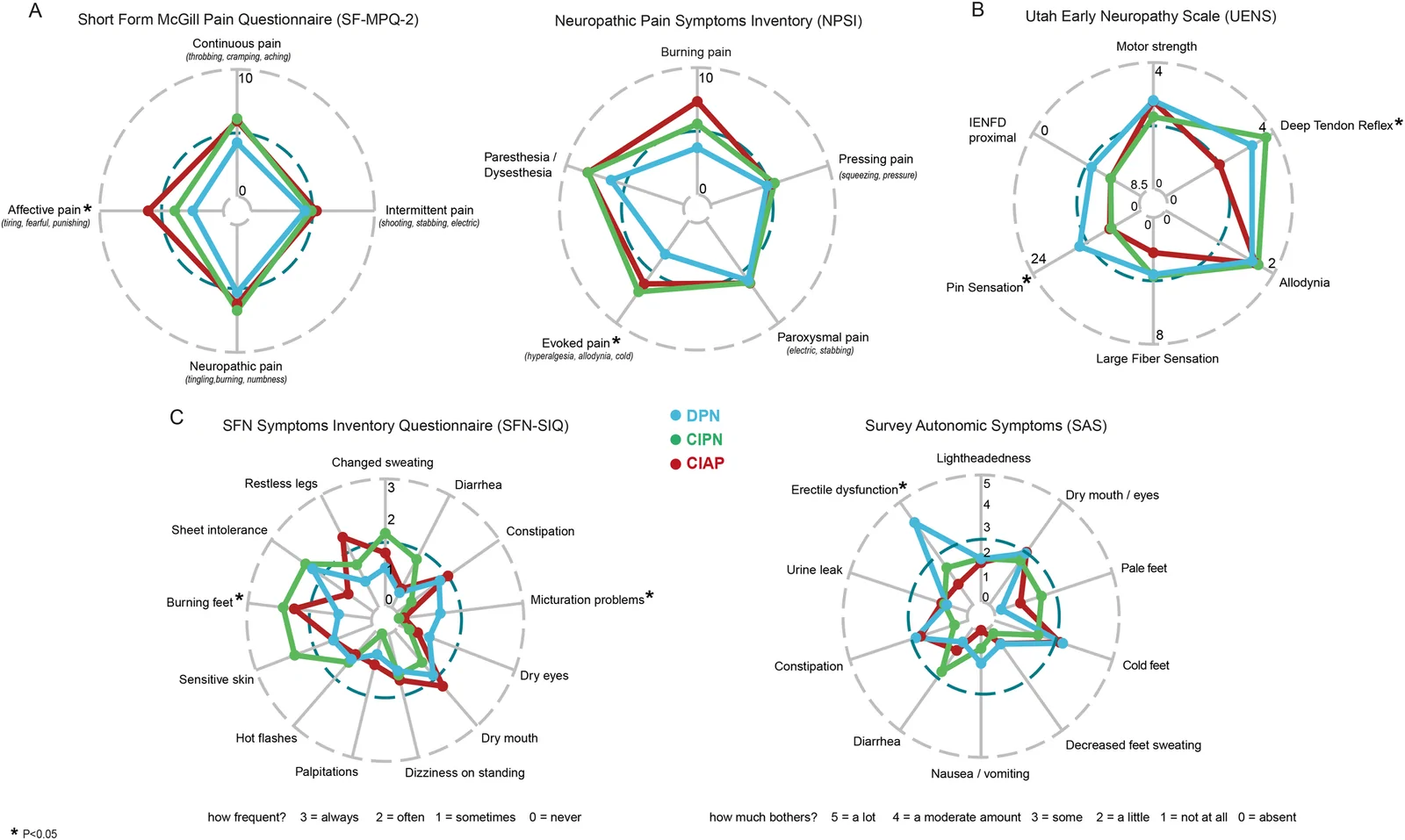
Baseline characteristics across polyneuropathy etiologies in pain symptoms, neuropathic symptoms, autonomic symptoms and neurological signs**.** Baseline differences across etiologies in **(A)** multidimensional pain (SF-MPQ-2) and neuropathic symptoms (NPSI); **(B)** neurological signs (UENS); and **(C)** autonomic symptoms (SFN-SIQ and SAS).

In contrast, neurological impairment was greater among patients with DPN. UENS total scores were higher in DPN compared with CIAP (20.3 vs. 13.3; ANOVA F = 3.62, *p* = 0.04). Subscore analyses showed that DPN had worse pin sensation, whereas CIAP had less large fiber involvement (large fiber sensation and deep tendon reflex) (Figure 2B). IENFD mean values were also lower in DPN compared with CIAP at both distal-leg (2.2 vs. 3.1 fibers/mm) and proximal-thigh (4.4 vs. 6.1 fibers/mm), although these differences were not significant.

Autonomic symptom burden was comparable across groups; however, item-level analyses identified differences in specific autonomic manifestations. Specifically, DPN had higher scores on erectile dysfunction (SAS-12: 4.0 vs. 2.25 (CIPN) and 1.2 (CIAP); *p* = 0.05) and micturition problems (SFN-SIQ-4: 1.0 vs. 0.0 (CIPN) and 0.1 (CIAP); *p* = 0.04) (Figure 2C). No significant between-group differences were observed in HR-QoL, sleep, or functioning.

### Clinical outcomes after SCS implantation in the overall cohort

Following SCS implantation, patients showed significant and sustained improvements across pain, autonomic symptoms, HR-QoL, sleep, and functioning (Table 2; Figure 3). Longitudinal analyses using linear mixed-effects models showed significant improvements over time across patient-reported outcomes, with the largest effects observed in pain-related measures.

**Table 2:** . Longitudinal changes in pain, neurological and wellbeing outcomes following SCS in the overall PPN cohort.

Outcome	[min, max]	Baseline (n = 30)	2 months (n = 30)	6 months (*n* = 26)	12 months (n = 21)	β [95% CI]	*P*-value
Pain							
VAS	[0,10]	8.6 (0.9)	2.4 (1.8)	2.6 (1.7)	2.0 (1.5)	−6.6 [-7.6 to -5.6]	<0.001
DN4	[0,10]	7.0 (1.3)	3.8 (2.3)	3.4 (2.0)	3.0 (2.3)	−4.0 [-5.3 to -2.7]	<0.001
NPSI	[0,100]	61.1 (20.9)	17.1 (15.6)	12.8 (11.9)	11.1 (14.9)	−50.0 [-37.2 to -62.7]	<0.001
SF-MPQ-2	[0,220]	115.5 (42.3)	31.5 (34.9)	27.0 (29.4)	23.0 (30.4)	−91.9 [-64.0 to -118.2]	<0.001
Autonomic							
SFN-SIQ	[0, 39]	13.5 (5.4)	8.7 (4.7)	8.5 (5.8)	7.3 (5.4)	−6.2 [−10.3 to −1.2]	0.001
SAS	[0, 60]	16.5 (8.6)	12.9 (8.4)	11.0 (6.1)	9.9 (6.0)	−6.6 [−11.1 to −0.7]	0.02
HR-QoL							
EQ-index	[0,1]	0.70 (0.06)	0.91 (0.10)	0.91 (0.10)	0.93 (0.10)	+0.23 [0.18 to 0.29]	<0.001
EQ-VAS	[0,100]	38.8 (18.0)	76.0 (10.6)	76.5 (10.0)	77.9 (9.7)	+39.1 [32.1 to 46.1]	<0.001
Sleep							
CPSI-3	[0,30]	18.4 (8.3)	3.3 (6.3)	4.3 (6.1)	3.5 (6.0)	−14.9 [-9.1 to -20.0]	<0.001
Functioning							
GAF	[0,100]	63.9 (9.4)	84.0 (7.6)	84.9 (6.2)	86.1 (5.5)	+22.2 [16.3 to 27.5]	<0.001
Neurological							
UENS	[0,42]	17.3 (6.5)	16.1 (6.0)	16.5 (5.2)	16.4 (5.4)	−0.9 [-5.1 to 3.5]	0.96
IENFD_proximal_ *(n* *=* *15)*	[0, ≥8.5]	5.0 (2.4)	-	-	4.8 (2.0)	−0.2 [-0.6 to 1.1]	0.60
IENFD_distal_ *(n* *=* *15)*	[0, ≥4.5]	3.1 (2.0)	-	-	3.1 (1.0)	−0.03 [-0.8 to 0.9]	0.92

Scale values for minimum and maximum ranges, as well as cutoffs defining normality, mild, and severe categories, are specified.

VAS, visual analog scale; DN4, douleur neuropathique-4; NPSI, neuropathic pain symptoms inventory; SF-MPQ-2, short-form McGill pain questionnaire-2; SFN-SIQ, small fiber neuropathy symptoms inventory questionnaire; SAS, survey of autonomic symptoms; HR-QoL, health-related quality of life; EQ, EuroQol; CPSI-3, 3-item chronic pain sleep inventory; GAF, global assessment of functioning; UENS, Utah early neuropathy scale; IENFD, intraepidermal nerve fiber density.

P-value: statistical significance (p < 0.05) for the time factor in a Linear mixed model.

**Figure 3:**
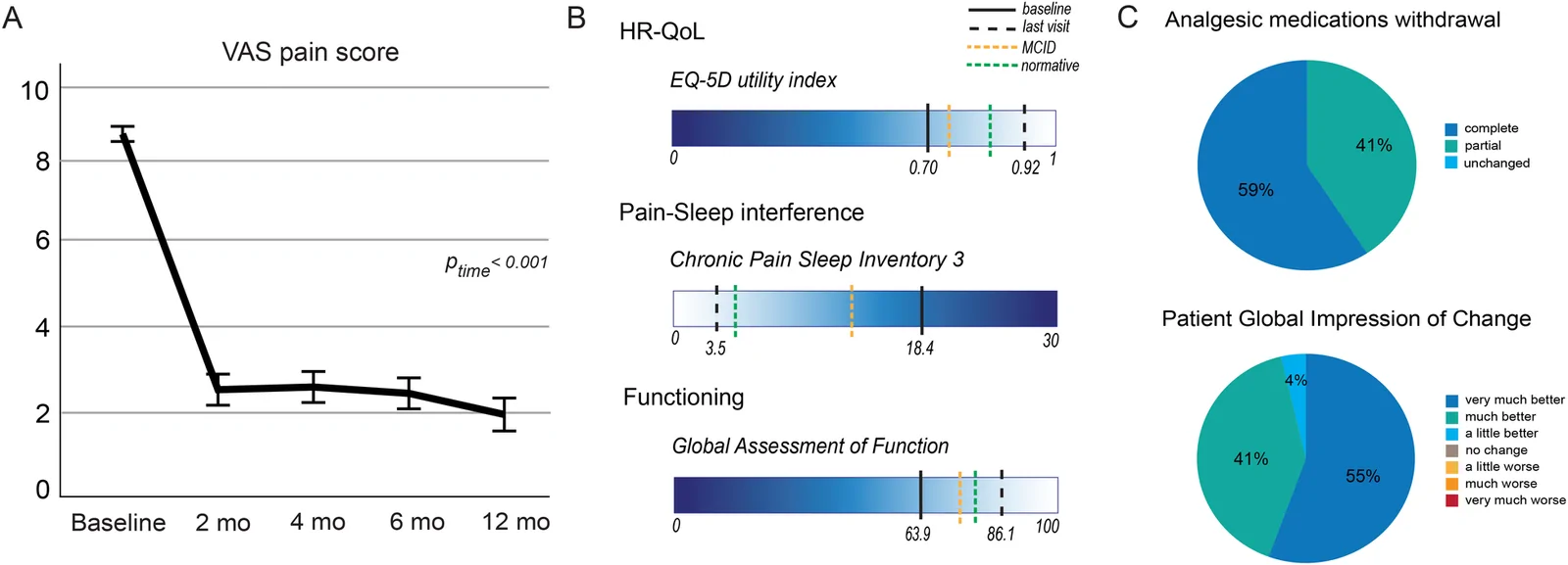
Pain intensity, health-related outcomes, and changes in use of analgesics and patient global impression following SCS. **(A)** Longitudinal changes in Visual Analog Scale pain score (mean ± sem); **(B)** Health-related (HR) outcomes: HR-Quality of life (EQ-5D utility index), Pain-Sleep interference (Chronic Pain Sleep Inventory – 3) and Functioning (Global Assessment of Function). Lines denote values at baseline, last visit, minimal clinical important difference (mcid) and normative values; **(C)** Distribution of analgesic medications use and Patient Global Impression of Change at last follow-up.

Pain intensity decreased rapidly after implantation and remained improved through 12 months. Mean VAS score decreased from 8.6 at baseline to 2.4 (2 months) and to 2.0 (12 months), corresponding to an approximate 75% reduction (*p* < 0.001) (Figure 3A). Similar improvements were observed in neuropathic pain symptoms, with NPSI decreasing from 61.1 to 11.1 (*p* < 0.001) substantially exceeding the MCID threshold of 8 points (NPSI) (25); and multidimensional pain symptoms, with SF-MPQ-2 decreasing from 115.5 to 23.0 (*p* < 0.001).

Autonomic symptom burden also improved following SCS. Mean SFN-SIQ scores decreased 46% from 13.5 at baseline to 7.3 at 12 months (*p* = 0.001), while SAS scores decreased 40% from 16.5 to 9.9 (*p* = 0.02), representing an average reduction of approximately 40% in autonomic symptom burden across etiologies. In this result, one must note that the SAS comprises exclusively autonomic questions, whereas the SFN-SIQ includes three sensory items. In addition, by 12 months, there was a significant reduction in the mean number of clinically relevant autonomic symptoms (SAS score ≥3), decreasing from 3.2 ± 2.1 to 1.8 ± 1.5 symptoms (*p* = 0.01). Item-level analyses demonstrated significant improvements from baseline to 12 months in specific autonomic-related symptoms. Within the SFN-SIQ, significant reductions were observed in sensitive skin, hot flashes, and dizziness on standing (all *p* < 0.01), with trends toward improvement in palpitations, altered sweating pattern, diarrhea, and dry mouth (*p* = 0.05). Within the SAS, significant reductions were observed in diarrhea and cold feet (*p* < 0.01), as well as nausea/bloating and lightheadedness (*p* < 0.05).

Additional health-related outcomes demonstrated significant improvement (Figure 3B). Pain-related sleep impairment decreased from 18.4 to 3.5 on CPSI-3 (*p* < 0.001), shifting from severe to mild impairment. Functional status improved, with GAF scores increasing from 63.9 to 86.1 (*p* = 0.001). HR-QoL improved substantially, with EQ-VAS increasing from 38.8 to 77.9 and EQ-index increasing from 0.70 to 0.93, corresponding to improvements largely exceeding the reported MCID of 0.05 and normative thresholds for age-matched Spanish populations (26).

Significant reduction in use of analgesics was also observed during follow-up: at the final assessment, 59% of patients had discontinued analgesic medications and the remaining 41% had reduced their use. PGIC and CGIC indicated a favorable overall response, with 96% of participants rated as “much improved” or “very much improved” at the final follow-up (Figure 3C).

Mean IENFD values remained stable among patients with available follow-up data (*n* = 15), with IENFD_proximal_ changing from 5.0 to 4.8 fibers/mm and IENFD_distal_ remaining unchanged at 3.1 fibers/mm. In the non-parametric analysis, although not statistically significant, positive changes outnumbered negative changes: IENFD_proximal_ increased in 8 patients and declined in 7, wheras IENFD_distal_ increased in 9, declined in 5 and remained equal in 1. Given that IENFD is expected to decline on average by approximately 11% annually in these PPN conditions (27), the overall stability of IENFD over 12 months suggests attenuation or stabilization of small-fiber degeneration following SCS.

Finally, we did not observe changes in neurological signs, including sensory and motor tests (UENS from 17.3 to 16.4; *p* = 0.96). There was only a significant improvement at the group level in the allodynia subscore (1.7 to 1.1 points; *p* = 0.05). Regarding stimulation modality, 25 of 30 patients (83%) selected the sub-perception modality (FAST^TM^), while 5 of 30 (17%) selected the combination modality (supra- and sub-perception, COMBO^TM^).

### Differences in clinical outcomes by PPN etiology

When analyses were stratified by neuropathy etiology, differences in the magnitude of response to SCS were observed across groups (Figures 4A,B). Although all etiologies demonstrated significant improvement over time, the reduction in pain-related outcomes was greatest in patients with DPN, followed by CIPN and CIAP. Across pain measures, estimated percentage reductions were approximately 85% in DPN, 75% in CIPN, and 70% in CIAP. The patient with Fabry disease experienced a 55% reduction in pain intensity (VAS) and reductions of approximately 35% in overall pain symptoms (SF-MPQ-2, from 80 to 52) and 65% in neuropathic symptoms (NPSI, from 45 to 16). The patient with amyloidosis also experienced a 55% reduction in pain intensity, with greater reductions of approximately 70%–80% in overall pain and neuropathic symptoms (SF-MPQ-2, from 155 to 29; NPSI, from 88 to 23).

**Figure 4:**
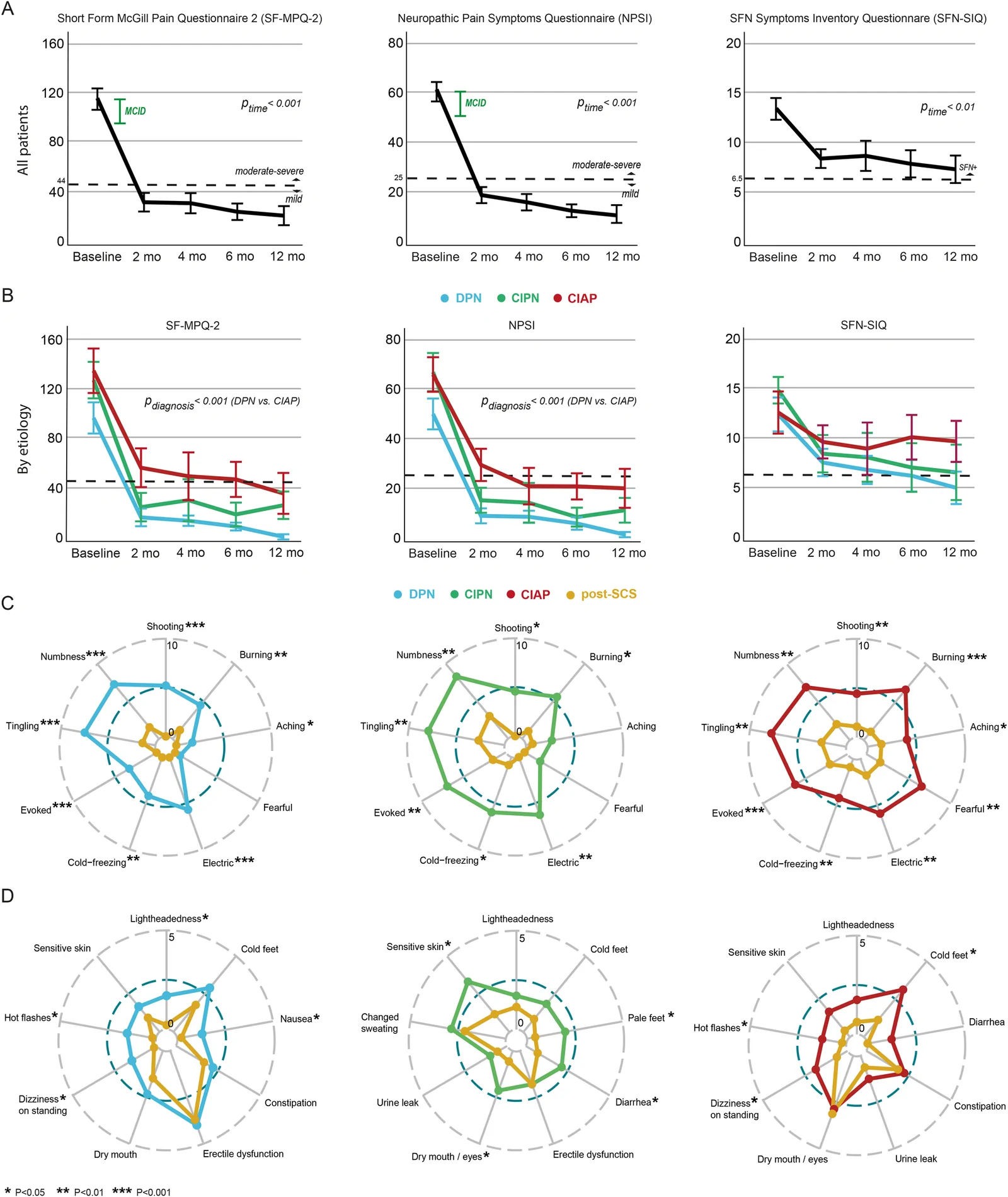
Longitudinal changes following SCS for multidimensional pain symptoms, neuropathic symptoms and autonomic symptoms, for all patients and stratified by etiology. Longitudinal changes in multidimensional pain symptoms (SF-MPQ-2), neuropathic symptoms (NPSI) and small-fiber neuropathy symptoms (autonomic and neuropathic) (SFN-SIQ) for **(A)** all patients; and **(B)** stratified by etiology; **(C)** Changes in selected pain and neuropathic descriptors (SF-MPQ-2 and NPSI) per etiology **(D)** Changes in selected autonomic descriptors (SAS and SFN-SIQ) per etiology. Colors: DPN (light blue), CIPN (green), CIAP (dark red), last follow-up post-SCS (gold).

Linear mixed-effects models demonstrated a significant effect of etiology on treatment response, with greater improvement in DPN compared with CIAP (group effect, *p* < 0.001). This pattern was consistent across VAS, DN4, NPSI, and SF-MPQ-2 scores. Domain-specific analyses of neuropathic pain questionnaires confirmed improvements across multiple symptom dimensions, including burning, paresthesia, numbness, and evoked pain, with differences in magnitude of change across etiologies (Figure 4C).

Sub-group analyses also revealed some heterogeneity in autonomic symptoms relief, both in terms of symptoms and magnitude (Figure 4D). In patients with DPN, improvements were significant in orthostatic and cardiovascular-related symptoms, including lightheadedness, hot flashes, and dizziness upon standing, as well as in nausea/bloating. In CIPN, reductions were significant in diarrhea, limb color changes (pale/blue feet), secretomotor symptoms (dry mouth/eyes), and skin sensitivity. In CIAP, improvements were significant in cold feet, hot flashes, and dizziness upon standing. The patients with Fabry disease and amyloidosis did not experience meaningful reductions in autonomic symptoms. Neurological signs remained largely unchanged across etiologies. The exception was allodynia, which showed significant reduction in DPN (from 1.75 to 0.75, *p* = 0.01), trend in CIPN (from 1.75 to 1.00, *p* = 0.11), but remained unchanged in CIAP (from 1.67 to 1.33).

Etiology-specific analyses of IENFD showed broadly similar patterns across subgroups, with overall stability observed across etiologies. Specifically, in patients with CIAP (*n* = 7), IENFD_proximal_ evolved from 6.3 to 6.1 and IENFD_distal_ from 3.6 to 3.2 fibers/mm; in DPN (*n* = 4), IENFD_proximal_ from 3.3 to 3.6 and IENFD_distal_ from 2.6 to 2.9 fibers/mm; and in CIPN (*n* = 3), IENFD_proximal_ from 3.7 to 3.1 and IENFD_distal_ from 2.5 to 2.6 fibers/mm.

The patient with Fabry disease showed a decline in IENFD_proximal_, from 6.8 to 5.8 fibers/mm, and an increase in IENFD_distal_, from 3.4 to 4.0 fibers/mm.

Because the longitudinal study by Khoshnoodi et al. observed some differences in the rate of IENFD decline according to etiology and biopsy site (27), we performed additional etiology-specific projections, presented in Figure 5. In that study, the estimated annual decline was greater in CIAP (16%–23%) than in DPN (6%–7%). As CIPN was not included in their cohort, and it remains unclear whether IENFD loss in CIPN reflects a transient injury following chemotherapy or an ongoing progressive decline (28), we did not assume an annual rate of IENFD decline for CIPN in our projections.

**Figure 5:**
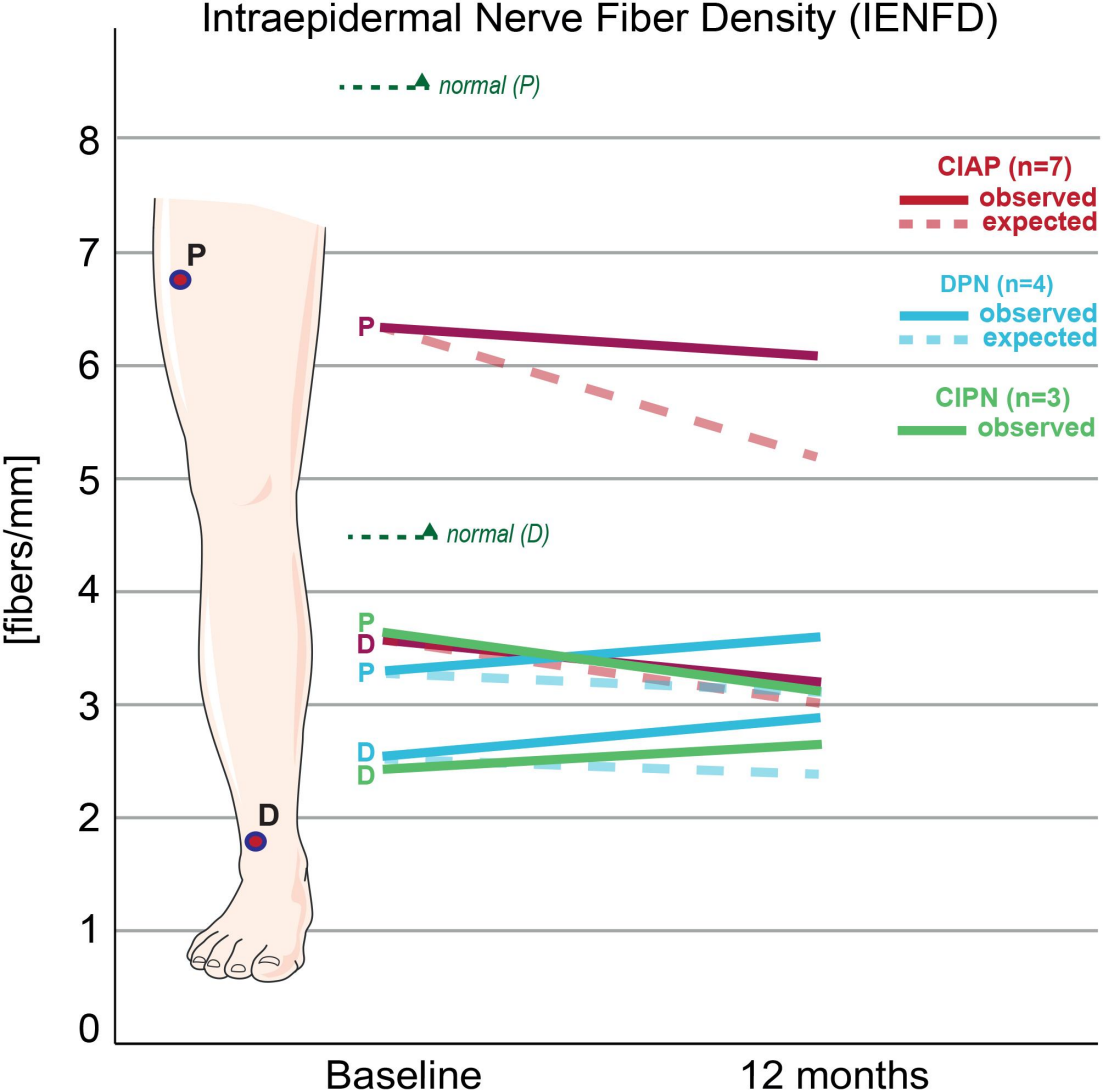
Changes in intraepidermal nerve fiber density (IENFD) following SCS, stratified by etiology**.** Biopsies were obtained from the distal leg (DL) and proximal thigh (PT). Observed changes (solid lines) are shown alongside the projected natural decline (dashed lines) derived from Khoshnoodi et al.,2016 (27). Annual decline rates were estimated at 23% (PT) and 16% (DL) for CIAP, 6% (PT) and 7% (DL) for DPN. No annual decline assumptions were made for CIPN.

No other clinically relevant differences in SCS response were observed among etiologies across measures of wellbeing and functional status.

## Discussion

Our results support the use of SCS as an effective therapy for relieving pain, as well as neuropathic and autonomic symptoms, and for improving sleep and other quality of life measures in patients with chronic polyneuropathy associated with diabetes, chemotherapy, or idiopathic etiologies whose pain is refractory to conventional pharmacological treatments. The observed outcomes were clinically meaningful, exceeding MCID thresholds and maintained at 6 and 12 months post-implantation. Furthermore, our findings extend the existing evidence supporting SCS in idiopathic and chemotherapy-induced PPN and provide additional insights into SCS-related aspects, including the use of cervical lead and low-frequency, sub-perception stimulation.

Overall, our findings are consistent with previous studies demonstrating that SCS is an effective treatment for painful PPN. The magnitude of pain relief and improvements in other health-related outcomes, including HR-QoL, sleep, and functioning, are comparable to those reported in recent studies in DPN (29, 30). Evidence for CIPN remains limited to case reports (15), although these support the effectiveness of SCS in this population. In contrast, evidence for CIAP has been lacking, despite this subtype potentially accounting for up to one-third of PPN cases. Our findings begin to fill this gap and may provide a basis for further investigation in this patient population.

Our baseline characterization revealed important differences across PPN etiologies that likely reflect underlying disease mechanisms. The shorter pain duration observed in CIPN may be explained by its more acute onset compared with the slowly progressive nature of CIAP and DPN, as well as potential delays in access to advanced therapies in the latter groups. The trend toward lower NPSI and SF-MPQ-2 scores in DPN suggest a less complex pain phenotype compared with CIAP and CIPN. Specifically, DPN was associated with lower levels of tingling, burning, and evoked pain, whereas CIAP demonstrated a greater affective pain component. Given that hyperalgesia, allodynia, and emotional distress are key features of central sensitization (31), our findings suggest a greater degree of central sensitization in CIAP compared with DPN. This may reflect not only disease-related mechanisms but also differences in patient experience. Patients with CIAP often undergo prolonged diagnostic processes with uncertainty regarding etiology, which may contribute to increased psychological burden and amplification of pain perception.

These baseline differences in pain phenotype may also have prognostic implications. For instance, lower pain-related fear in DPN might partially explain the higher withdrawal rate prior to implantation (35% in DPN vs. 0% in CIAP and CIPN) as well as the greater response to SCS observed in this group, both in the short term (100% trial success vs. 77% in other groups) and long term. Conversely, the higher levels of evoked pain in CIAP may reflect greater central sensitization and impaired spinal pain inhibitory mechanisms (31), which are known to play a role in the mechanism of action of SCS (32). These findings support the importance of comprehensive baseline characterization to inform patient selection and set realistic expectations for SCS outcomes.

Improvements in autonomic symptoms may have further contributed to enhanced patient wellbeing and quality of life. Although the effects of SCS on autonomic function have been described in other conditions, including peripheral vascular disease (PVD) and spinal cord injury (SCI), they remain largely unexplored in PPN. There is no established MCID for SFN-SIQ and SAS, but the observed ∼40% reduction appears clinically meaningful based on the IMMPACT recommendations on interpreting outcomes in pain clinical research (33). Across etiologies, SCS appeared to improve a range of cardiovascular- and sudomotor-related symptoms, including lightheadedness, dizziness upon standing, cold/pale feet, hot flashes, skin hypersensitivity, and altered sweating pattern, which may reflect improved vasomotor regulation, peripheral perfusion and thermoregulatory activity as previously described in individuals with PVD (34) and SCI (35). However, the effect on erectile dysfunction, particularly prevalent and bothersome in DPN, was limited. Although positive evidence on sexual function has been reported in SCI (36), further optimization of SCS may be required to specifically target this impairment. The observed gastrointestinal improvements in nausea/bloating and diarrhea can be particularly relevant for DPN and CIPN, respectively. Diarrhea represents a frequent and burdensome side effect from chemotherapy (37).

Greater baseline neurological impairment in DPN compared with CIAP, as shown by UENS and IENFD, indicates more severe involvement of both small and large fibers. As these differences were not explained by pain duration, they may reflect a more aggressive neurodegenerative process in DPN, consistent with previous findings (27). A notable observation was the discrepancy between SCS-induced improvements in neurological symptoms and signs. While symptoms such as burning and numbness improved substantially (∼70%), associated signs such as pin or large fibers sensation showed limited change overall. This likely reflects the distinction between rapid modulation of neural activity and central pain processing vs. the slower processes of axonal regeneration and remyelination required for structural recovery (38). Results in this regard in the literature are mixed. While Petersen et al. reported meaningful neurological improvements, mostly sensory, after 10 kHz SCS in 52/85 DPN subjects (13), Koetsier et al. did not observe changes using DRG-S in 7 DPN subjects (39). These discrepancies may also reflect differences in neuromodulation techniques, stimulation parameters and/or neurological assessment methods.

Our IENFD findings, including a higher proportion of increases and a lower-than-expected mean overall decline, suggest that SCS may have some effect on small-fiber integrity. However, these effects appear modest, and larger controlled studies are needed to establish causality. The variability observed across etiologies, biopsy sites, and individual patients is consistent with that reported in other longitudinal IENFD studies (27, 28, 40). The stabilization or modest increase observed in DPN is also similar to findings reported by Koetsier et al. (39). The average stabilizing effect in CIAP exceeds the pronounced IENFD decline observed by Khoshnoodi et al. (27). In CIPN, long-term IENFD longitudinal data remain limited, and it remains unclear whether nerve injury occurs predominantly shortly after chemotherapy or whether fiber loss continues to progress over time (41). Therefore, given the small sample and uncertain natural history of IENFD in CIPN, our results in this subgroup require further data for adequate interpretation.

Approximately 40% of patients presented with upper limb involvement, consistent with the length-dependent nature of polyneuropathy. This highlights the importance of comprehensive clinical assessment and supports the consideration of cervical lead placement when indicated. The preference for sub-perception stimulation (83% vs. 17%) likely reflects the high prevalence of paresthesia/dysesthesia in this population. The use of low-energy stimulation paradigms may be particularly advantageous, reducing device recharge burden and minimizing potential adverse effects.

The main limitations of this study include the relatively small sample size and the heterogeneity of PPN etiologies, which may limit subgroup comparisons. Ongoing data collection will help strengthen these findings. Additionally, more comprehensive neurophysiological assessments would have provided further insight into mechanisms of action. Completion of 12-month IENFD analyses is also pending.

## Data Availability

The raw data supporting the conclusions of this article will be made available by the authors, without undue reservation.
